# Transverse Relaxation Anisotropy of the Achilles and Patellar Tendon Studied by MR Microscopy

**DOI:** 10.1002/jmri.28095

**Published:** 2022-02-05

**Authors:** Benedikt Hager, Markus M. Schreiner, Sonja M. Walzer, Lena Hirtler, Vladimir Mlynarik, Andreas Berg, Xeni Deligianni, Oliver Bieri, Reinhard Windhager, Siegfried Trattnig, Vladimir Juras

**Affiliations:** ^1^ Institute for Clinical Molecular MRI in the Musculoskeletal System Karl Landsteiner Society Vienna Austria; ^2^ Department of Orthopedics and Trauma‐Surgery Medical University of Vienna Austria; ^3^ Center for Anatomy and Cell Biology, Division of Anatomy Medical University of Vienna Austria; ^4^ Center for Medical Physics and Biomedical Engineering Medical University of Vienna Vienna Austria; ^5^ Division of Radiological Physics, Department of Radiology University of Basel Hospital Basel Switzerland; ^6^ Department of Biomedical Engineering University of Basel Allschwil Switzerland; ^7^ Basel Muscle MRI, Department of Biomedical Engineering University of Basel Allschwil Switzerland; ^8^ High Field MR Centre, Department of Biomedical Imaging and Image‐guided Therapy Medical University of Vienna Austria; ^9^ CD Laboratory for Clinical Molecular MR Imaging Vienna Austria; ^10^ Austrian Cluster for Tissue Regeneration Ludwig Boltzmann Institute for Experimental and Clinical Traumatology Vienna Austria

**Keywords:** magic angle artifact, magic angle effect, fiber‐to‐field angle dependence, T_2_* anisotropy, T_2_* mapping, MR microscopy, tendon

## Abstract

**Background:**

T_2_* anisotropy affects the clinical assessment of tendons (magic‐angle artifact) and may be a source of T_2_*‐misinterpretation.

**Purpose:**

To analyze T_2_*‐anisotropy and T_2_*‐decay of Achilles and patellar tendons in vitro at microscopic resolution using a variable‐echo‐time (vTE) sequence.

**Study Type:**

Prospective.

**Specimen:**

Four human Achilles and four patellar tendons.

**Field Strength/Sequence:**

A 7 T MR‐microscopy; 3D‐vTE spoiled‐gradient‐echo‐sequence (T_2_*‐mapping).

**Assessment:**

All tendons were measured at 0° and 55° relative to B_0_. Additional angles were measured for one Achilles and one patellar tendon for a total of 11 angles ranging from 0° to 90°. T_2_*‐decay was analyzed with mono‐ and bi‐exponential signal fitting. Mono‐exponential T_2_*‐values (T_2_*_m_), short and long T_2_*‐components (T_2_*_s_, T_2_*_l_), and the fraction of the short component F_s_ of the bi‐exponential T_2_*‐fit were calculated. T_2_*‐decay characteristics were compared with morphological MRI and histologic findings based on a region‐of‐interest analysis.

**Statistical Tests:**

Akaike information criterion (AIC_C_), *F*‐test, and paired *t*‐test. A *P* value smaller than the α‐level of 0.05 was considered statistically significant.

**Results:**

T_2_*_m_‐values between fiber‐to‐field angles of 0° and 55° were increased on average from T_2_*_m_ (0°) = 1.92 msec to T_2_*_m_ (55°) = 29.86 msec (15.5‐fold) in the Achilles and T_2_*_m_ (0°) = 1.46 msec to T_2_*_m_ (55°) = 23.33 msec (16.0‐fold) in the patellar tendons. The changes in T_2_*_m_‐values were statistically significant. For the whole tendon, according to *F*‐test and AIC_C_, a bi‐exponential model was preferred for angles close to 0°, while the mono‐exponential model tended to be preferred at angles close to 55°.

**Conclusion:**

MR‐microscopy provides a deeper insight into the relationship between T_2_*‐decay (mono‐ vs. bi‐exponential model) and tendon heterogeneity. Changes in fiber‐to‐field angle result in significant changes in T_2_*‐values. Thus, we conclude that awareness of T_2_*‐anisotropy should be noted in quantitative T_2_*‐mapping of tendons to avoid T_2_*‐misinterpretation such as a false positive detection of degeneration due to large fiber‐to‐field angles.

**Evidence Level:**

2

**Technical Efficacy:**

Stage 2

Quantitative T_2_* mapping of highly ordered collagen‐rich tissues such as tendons are associated with considerable challenges. Although it has been shown in recent years that T_2_* mapping has great potential to detect degenerations in these tissues noninvasively and even earlier than conventional morphological MRI,^1^ many interpretative aspects of this method are not yet fully understood. A prominent property is the influence of the orientation of collagen fibers in relation to the static magnetic field on the transverse relaxation times (T_2_ and T_2_*). This is due to the structural composition of collagen in which the water molecules surrounding the collagen fibers move slowly and have a preferred orientation (the ensemble‐averaged intramolecular <H–H> vector is parallel to the fiber), and the translational motion is restricted to preferred directions.^2^, ^3^, ^4^ In MRI, this orientational restriction of the water molecules causes residual dipolar coupling of protons leading to orientation dependent T_2_ and T_2_* values, often simply referred to as the “magic angle effect”.^3^, ^5^, ^6^ Due to the dipolar interaction of protons in collagen, the T_2_ and T_2_* values differ at any angle, not just the “magic angle” of 54.7°, and inherently cannot be suppressed.^3^ More detailed information with some theoretical background can be found in the Supplementary Section: “Theory ‐ Dipolar intramolecular interaction of protons.”.

Although it has been shown that T_2_* mapping using ultrashort echo time (UTE) has the potential to detect degenerative changes and other tissue abnormalities in the tendon,^1^, ^7^ the orientation dependence of transverse relaxation times has not been taken into consideration in these studies. In previous studies, however, it has been shown that the T_2_ and T_2_* anisotropy can be very considerable.^8^, ^9^, ^10^, ^11^, ^12^

Another interesting feature of tendon imaging is the observed multiexponential character of the transverse relaxation decay (T_2_/T_2_*), which is found in nonlocalized spectroscopic MR measurements,^8^, ^13^ where the signal obtained is a mixture of signals stemming from the entire object, and for example, in MRI experiments with a voxel size of 0.2 × 0.2 × 2 mm that is typical for high‐resolution whole‐body MRI.^11^

While there may be several T_2_/T_2_* components in the tendon tissue, as suggested by nonlocalized spectroscopic MR measurements,^8^, ^13^, ^14^ the observed T_2_* decay in tendinous tissue can often be well described with a bi‐exponential function, with the short and long components typically assigned to “bound” and “free water pools,” respectively.^1^, ^7^, ^15^, ^16^

These observations, however, were made in prior studies that used comparably low spatial resolution in their imaging experiments,^1^, ^7^ for example, 0.63 × 0.63 × 3 mm.^7^ At these spatial resolutions, it was not possible to resolve and analyze individual sub‐tissues. Thus, these studies and the interpretation of their results were based on the assumption that tendons represent a homogenous tissue. In reality, however, the anatomical structure of the tendon^17^, ^18^ is quite complex, with a dominant collagen fascicle component, but also a nonfascicle component, that includes the endotenon, which acts as an inter‐dispersion between the collagen fascicles and contains blood, lymph vessels, and nerves.^18^ To be able to differentiate these different sub‐tissues of the tendon and to be able to visualize and analyze them independently, resolutions in the range of the microscopic scale (<100 μm) are necessary.

Previous multicomponent T_2_ and T_2_* analyses performed at low spatial resolution could not take into account the heterogeneity of the tissue.^1^, ^3^, ^7^, ^8^, ^10^, ^11^, ^13^, ^19^ However, tissue heterogeneity has already been suggested as a possible factor influencing multicomponent T_2_ decay.^8^, ^20^

It is commonly assumed that UTE sequences are required to obtain sufficient signal from a tendon for adequate MR parameter mapping. However, in order to achieve microscopic resolution simultaneously, a classical radial center‐out UTE sequence^21^, ^22^ might not be the ideal choice due to its characteristic of undersampling the outer k‐space region and its challenge to satisfy the Nyquist criterion for large matrix sizes.

Instead, a Cartesian 3D variable echo time (vTE) sequence^23^ might be better suited for such an experiment, since it has considerable advantages over a radial UTE sequence in terms of k‐space sampling efficiency under the condition that the Nyquist criterion is fulfilled, robustness, image quality and acquisition time, even if it does not achieve the short echo times that common radial UTE sequences can offer.^23^

In a previous study on human menisci, it was shown that the combination of ultra‐high field strength 7 T, an MR microscopy system and a 3D variable echo time sequence meets these requirements for ultra‐short echo times and microscopic resolution^24^ and as such represents an ideal, unique setup for the angle‐dependent MR examination of tendons and their respective T_2_* characteristics, which we wish to demonstrate in this study.

The objectives of this study are as follows:

1. To investigate in vitro the T_2_* anisotropy of tendons in their maxima and minima of dipolar interaction and additionally in 10° increments from 0° to 90°.
2. To investigate the T_2_* decay characteristics in different fiber‐to‐field angles.

## Methods and Materials

### Sample Preparation

Four Achilles and four patellar tendon specimens out of four unpaired lower extremities from four body donors (mean age: 87 years, 2 male, 2 female) were obtained from the Center for Anatomy and Cell Biology of our university (LH). Only tendons from body donors that were macroscopically intact and showed no signs of rupture or other pathological changes were used. This study was approved by the local ethics committee.

For the MRI experiments, the mid‐sections of the tendons were prepared with a length of approximately 25 mm. They were each positioned in the center of a plastic sphere (30 mm diameter) on a plastic cross, where they were fixed with a surgical thread to avoid any kind of movement during the measurement. Subsequently, the sphere was filled with physiological saline solution.^24^

### μMRI Experiment

The MR microscopy (μMRI) measurements were performed on a 7 T scanner (Siemens Healthineers, Erlangen, Germany) using a MR microscopy system with a gradient strength of 750 mT m^−1^ (RRI, Billerica, Massachusetts, USA) and a 39 mm diameter volume resonator (Rapid Biomedical, Würzburg, Germany).^25^

After harvesting the tendons, each tendon was carefully prepared and frozen at −80°. They were then thawed before the MR experiments and measured at room temperature. All tendons were measured at fiber‐to‐field angles, which refer to the maximum and the minimum of dipolar interaction, that is, at angles of 0° and 55°, respectively. Furthermore, one Achilles and one patellar tendon were measured at a total of 11 fiber‐to‐field angles: 0°, 10°, 20°, 30°, 40°, 50°, 55°, 60°, 70°, 80°, 90°. The four Achilles and four patellar tendons are subsequently referred to as AT1, AT2, AT3, AT4, PT1, PT2, PT3, and PT4. AT4 and PT4 were the tendons measured at 11 angles.

T_2_* mapping and a morphological MRI protocol in an axial image plane were performed. For vTE T_2_* mapping,^23^ interleaved echo trains were used and reordered online on the console using the scripting framework IceLuva.^26^ The protocol was 40 echo times (TEs): 0.66–51.62 msec; TR = 80 msec; in‐plane pixel size = 98 × 98 μm^2^; slice thickness = 400 μm; number of slices = 72; FOV = 30 × 30 mm^2^; matrix size = 320 × 320; flip angle = 16°; pixel bandwidth = 220 Hz; TA = 96 min (per angle).

The morphological MRI protocol included a sagittal T_2_‐weighted fast spin echo (FSE) sequence, which was used to show different expressions of the signal intensity with different echo times: TEs = 6.6, 13, 40, 53 msec; TR = 2800 msec, in‐plane pixel size = 59 × 59 μm^2^; slice thickness = 400 μm; FOV = 30 × 30 mm^2^; matrix size = 512 × 512 and number of averages = 12.

### Analyzing T_2_ * Decay

The mono‐ and bi‐exponential T_2_* decay were calculated in‐house in IDL 6.3 (Interactive Data Language, Research Systems, Inc, Boulder, CO) as described in the literature.^24^ The mono‐exponential T_2_* values (T_2_*_m_) were calculated using a three‐parameter model fit. Bi‐exponential fitting was performed using a five‐parameter model fit. A short component of T_2_* (T_2_*_s_) and a long component of T_2_* (T_2_*_l_) were obtained. In addition, a short component fraction F_s_ (%) and a long component fraction F_l_ (%) were calculated. Only the short component fraction will be presented in the results as the long component is simply F_l_ = 100 ‐ F_s_. The component fraction that is larger in percentage terms will be referred to as the more pronounced component below.

In Tables 1, 2, 3, 4, the column “Bi‐comp.”, in (%), indicates how many of the slice‐wise evaluated T_2_* decays were to be considered following a bi‐exponential decay pattern. We considered those to be bi‐exponential, where the corresponding bi‐exponential fit yielded physically meaningful results.^27^ For this, we determined that the calculated T_2_* components must be positive and also the calculated short and long components of T_2_* must result in nonidentical T_2_* components. Furthermore, for both the *F*‐test and the AIC_C_, the bi‐exponential model should be preferred over the mono‐exponential model.

**TABLE 1:** T_2_* Decay Analysis at a Fiber‐to‐Field Angle of 0° Using 30 Consecutive Slices From Each Tendon

Tendon 0° (slice wise)	T_2_*_m_ ± SD (msec)	T_2_*_s_ ± SD (msec)	T_2_*_l_ ± SD (msec)	F_s_ ± SD (%)	Bi‐comp (%)
AT1	0.90 ± 0.04	0.66 ± 0.02	7.30 ± 0.52	94.38 ± 0.67	100
AT2	1.08 ± 0.09	0.72 ± 0.07	12.58 ± 0.87	92.20 ± 0.88	100
AT3	3.44 ± 0.19	1.29 ± 0.06	14.17 ± 0.69	76.57 ± 0.95	100
AT4	2.26 ± 0.15	1.44 ± 0.13	10.88 ± 0.74	84.59 ± 0.61	100
PT1	1.50 ± 0.48	0.69 ± 0.05	9.04 ± 1.08	87.09 ± 3.50	100
PT2	1.79 ± 0.72	0.60 ± 0.11	9.45 ± 0.51	85.30 ± 5.33	100
PT3	1.30 ± 0.16	0.83 ± 0.07	10.20 ± 0.68	89.75 ± 1.72	100
PT4	1.25 ± 0.22	0.68 ± 0.07	10.12 ± 0.77	89.41 ± 2.51	100

T_2_*_m_ = monoexponential T_2_*; T_2_*_s_ = short component of bi‐expo. T_2_*; T_2_*_l_ = long component of T_2_*; F_S_ = short component fraction of bi‐expo. T_2_*; Bi‐comp. = percent of slices that can be considered preferentially bi‐exponential (as described in Methods and Materials section).

**TABLE 2:** T_2_* Analysis At a Fiber‐to‐Field Angle of 55°

Tendon 55° (slice wise)	T_2_*_m_ ± SD (msec)	T_2_*_s_ ± SD (msec)	T_2_*_l_ ± SD (msec)	F_s_ ± SD (%)	Bi‐comp. (%)	*t*‐test *P* value
AT1	18.47 ± 1.22	(3.71 ± 1.09)	(22.57 ± 5.45)	(13.14 ± 5.88)	96.66	<0.001
AT2	34.14 ± 2.34	–	–	–	0	<0.001
AT3	38.61 ± 2.89	–	–	–	0	<0.001
AT4	28.23 ± 1.46	–	–	–	0	<0.001
PT1	21.17 ± 2.42	–	–	–	0	<0.001
PT2	19.80 ± 1.41	(1.63 ± 0.28)	(18.26 ± 0.89)	(5.75 ± 1.12)	20	<0.001
PT3	26.85 ± 2.06	(1.11 ± 0.15)	(26.40 ± 2.09)	(5.08 ± 1.32)	23.33	<0.001
PT4	25.49 ± 2.64	(0.93 ± 0.24)	(24.08 ± 1.30)	(6.76 ± 1.50)	43.33	<0.001

Thirty consecutive axial slices from the center of each tendon sample were evaluated, corresponding to the 30 slices in the 0° measurement.

T_2_*_m_ = monoexponential T_2_*; T_2_*_s_ = short component of bi‐expo. T_2_*; T_2_*_l_ = long component of T_2_*; F_S_ = short component fraction of bi‐expo. T_2_*; Bi‐comp. = percent of slices that can be considered preferentially bi‐exponential (as described in Methods and Materials section). *t*‐test *P* value refers to the statistical comparison between T_2_*_m_ values of 0° (Table 1) and 55° (Table 2) for the individual tendons.

**TABLE 3:** The Results of AT4 Measurements at 11 Fiber‐to‐Field Angles Are Presented. For Each Angle, 30 Slices Were Analyzed

Angle (°)	T_2_*_m_ (msec)	T_2_*_s_ (msec)	T_2_*_l_ (msec)	F_s_ (%)	Bi‐comp. (%)
0	2.26 ± 0.15	1.44 ± 0.13	10.88 ± 0.74	84.59 ± 0.61	100
10	2.45 ± 0.15	1.64 ± 0.11	12.40 ± 0.75	85.50 ± 0.74	100
20	3.43 ± 0.20	2.22 ± 0.12	11.99 ± 0.46	81.04 ± 1.09	100
30	5.99 ± 0.31	3.50 ± 0.17	10.60 ± 0.48	59.62 ± 3.70	100
40	13.19 ± 0.49	1.28 ± 0.16	13.83 ± 0.55	8.29 ± 0.50	100
50	28.21 ± 0.50	‐	‐	‐	0
55	28.23 ± 1.46	‐	‐	‐	0
60	37.93 ± 1.98	‐	‐	‐	0
70	17.24 ± 0.66	‐	‐	‐	0
80	8.88 ± 0.31	(0.76 ± 0.09)	(8.98 ± 0.36)	(5.88 ± 1.71)	23.3
90	7.71 ± 0.23	(6.86 ± 0.54)	(19.23 ± 7.40)	(86.21 ± 18.84)	40

T_2_*_m_ = monoexponential T_2_*; T_2_*_s_ = short component of bi‐expo. T_2_*; T_2_*_l_ = long component of T_2_*; F_S_ = short component fraction of bi‐expo. T_2_*; Bi‐comp. = percent of slices that can be considered preferentially bi‐exponential (as described in Methods and Materials section).

**TABLE 4:** The Results of the PT4 Measured at 11 Fiber‐to‐Field Angles

Angle (°)	T_2_*_m_ (msec)	T_2_*_s_ (msec)	T_2_*_l_ (msec)	F_s_ (%)	Bi‐comp. (%)
0	1.25 ± 0.22	0.68 ± 0.07	10.12 ± 0.77	89.41 ± 2.51	100
10	1.97 ± 0.34	1.07 ± 0.10	11.19 ± 0.59	84.20 ± 2.40	100
20	3.27 ± 0.48	1.57 ± 0.11	12.53 ± 1.22	77.35 ± 3.37	100
30	4.92 ± 0.64	2.15 ± 0.09	14.01 ± 1.40	69.01 ± 4.94	100
40	9.71 ± 0.98	3.47 ± 0.32	13.82 ± 1.29	34.26 ± 6.17	100
50	15.34 ± 1.16	3.38 ± 0.47	19.91 ± 1.98	19.95 ± 3.14	100
55	25.49 ± 2.64	(0.93 ± 0.24)	(24.08 ± 1.30)	(6.76 ± 1.50)	43.3
60	27.74 ± 2.78	(1.27 ± 0.67)	(27.58 ± 2.07)	(3.90 ± 2.03)	60
70	13.25 ± 0.60	3.02 ± 0.72	14.95 ± 0.88	12.61 ± 2.57	100
80	7.92 ± 0.69	3.87 ± 0.28	12.43 ± 0.80	46.66 ± 7.24	100
90	7.17 ± 0.64	3.96 ± 0.29	13.12 ± 1.18	57.06 ± 6.15	100

T_2_*_m_ = monoexponential T_2_*; T_2_*_s_ = short component of bi‐expo. T_2_*; T_2_*_l_ = long component of T_2_*; F_S_ = short component fraction of bi‐expo. T_2_*; Bi‐comp. = percent of slices that can be considered preferentially bi‐exponential (as described in Methods and Materials section).

### ROI Analysis

Evaluation of T_2_* relaxation times was performed using a manual region‐of‐interest (ROI) analysis. For each tendon, ROIs covering the entire axial slice(s) as well as ROIs containing selected substructures were placed. ROIs covering the entire axial slice of the respective tendons were used to evaluate the T_2_* characteristics of the whole tendon.

Another ROI analysis was performed in which three ROIs were selected for the Achilles tendon and two ROIs were selected for the patellar tendon and examined at 11 different angles. For the Achilles tendon, as shown in Fig. 3c,d, the ROI (red) was drawn in a nonfascicular part of the tendon, while the other two ROIs were drawn in the polygonally shaped fascicles (blue and yellow ROIs). For the patellar tendon, as shown in Fig. 4c,d, the ROI (purple) was drawn in the nonfascicular part of the tendon and the ROI (orange) was drawn in the fascicle part. The same ROI was drawn as best as possible in the same slice but at different angles, using the contrast between the nonfascicular tissue and the fascicles as landmarks for selection.

In this way, it may be possible to elucidate the contribution of partial volume effects to the bi‐exponential decay characteristics, previously observed in studies with insufficient spatial resolution,^1^, ^7^ and to independently map the relaxation times of different substructures of human tendons. ROIs, that cover the entire slice, can be used to explain the bi‐exponential T_2_* characteristics known from whole‐body quantitative T_2_* mapping.^1^, ^15^ For slice‐wise analysis, 30 consecutive slices from the midsection of each tendon sample were used.

### Histological Analysis

The MR measurement was followed by histological assessment of the tendon samples. The samples were processed as previously described.^24^ They were fixed with neutral‐buffered 4% formaldehyde and embedded in paraffin after decalcification. After deparaffinization 2.5‐μm serial slices were stained with Hematoxylin–Eosin (HE), used for morphological overview and Safranin O (SO) staining was used to gain further information on the zonal distribution of glycosaminoglycans. Picrosirius Red (PSR) staining was used for the detection of collagen under the light microscope.^24^

### Statistical Analyses

A paired *t*‐test was performed to compare T_2_*_m_ values of the 0° and 55° fiber‐to‐field angle measurement. A *P* value less than the α‐level of 0.05 was considered statistically significant.

To answer the question which model can be regarded as the preferred one for each T_2_* decay (i.e., mono‐ or bi‐exponential model) a model test (*F*‐test) and an information criterion (small sample bias corrected Akaike information criterion [AIC_C_]) were used.^24^, ^28^, ^29^

Both the *F*‐test and the AIC_C_ provide the means for model selection. They take into account the number of degrees of freedom and the echo times (data points) used and allow a statistical assessment of which of the two models (i.e., the mono‐ or the bi‐exponential model) is the preferred one. Thus, both are useful in avoiding either overfitting or underfitting of the data.

For the AIC_C_, the model with the minimum AIC_C_ value is considered the preferred model, while for the F‐test, a *P* < 0.05 was considered statistically significant, which in such a case indicates that the more complex model (i.e., the bi‐exponential model) is the statistically better model.

## Results

The mono‐exponential T_2_* values (T_2_*_m_) of the Achilles tendons were 11‐fold to 32‐fold higher at a fiber‐to‐field angle of 55° than at a fiber‐to‐field angle of 0° (Tables 1 and 2). Similarly, the mono‐exponentially calculated T_2_* values (T_2_*_m_) of the patellar tendons were 11‐fold to 21‐fold higher at a fiber‐to‐field angle of 55° compared with a fiber‐to‐field angle of 0°. The difference between the 0° and 55° measurement were statistically significant for all tendons (Table 2).

At an angle of 0°, a clear bi‐exponential decay was observed for all tendons according to AIC_C_ and *F*‐test. The T_2_* values of the short component (T_2_*_s_) were on average T_2_*_s_ = 0.86 msec and the long component (T_2_*_l_) was on average T_2_*_l_ = 10.47 msec. The short component was the more pronounced and had an average short component ratio (F_s_) of F_s_ = 87.41% at this angle (Tables 1 and 2).

**FIGURE 1:**
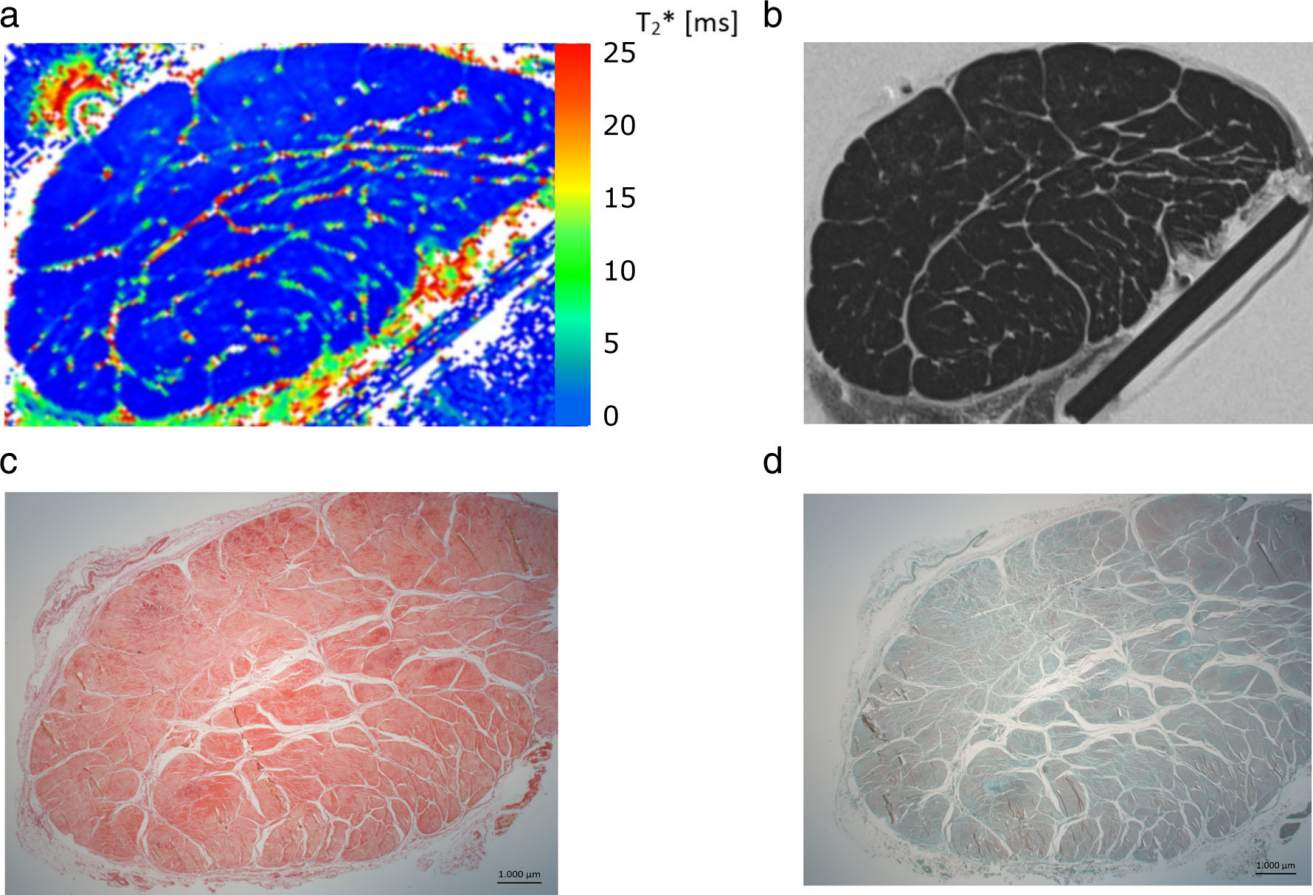
Representative mono‐exponentially calculated T_2_* map (a) and corresponding T_2_‐weighted image (TE = 6.6 msec) (b) of AT2 measured at 0°, and histological comparison with Picrosirius Red for collagen (c) and Safranin O for glycosaminoglycan staining (d) (magnification ×10). The polygon‐shaped collagen fascicles^30^ are clearly visible in all images and are surrounded by the endotenon.

Figure 1 shows the T_2_*_m_ map and morphological T_2_‐weighted image at 0° as well as the histological imaging by Picrosirius Red and Safranin O staining for AT2. The fascicle bundles displayed low T_2_* values in the T_2_* map and low signal intensity values in the morphological T_2_‐weighted image. The endotenon and other noncollagenous sub‐tissues presented higher T_2_* values and hyperintense signal.

Table 2 shows the results of the mono and bi‐exponential analysis. For AT2, AT3, AT4, and PT1, the mono‐exponential model was superior for all slices according to *F*‐test and AIC_C_. In contrast, for AT1, PT2, PT3, and PT4, the situation was more complex. For some slices, the bi‐exponential fitting was superior while for others the mono‐exponential fitting was preferred according to AIC_C_ and *F*‐test. For PT4, for example, 13 of 30 slices (43.33%) showed a preferential bi‐exponential T_2_* decay. The short component of T_2_* of these 13 slices was on average T_2_*_s_ = 0.93 msec, while the long component was T_2_*_l_ = 24.08 msec and the short component fraction was *F*_s_ = 6.76%.

**FIGURE 2:**
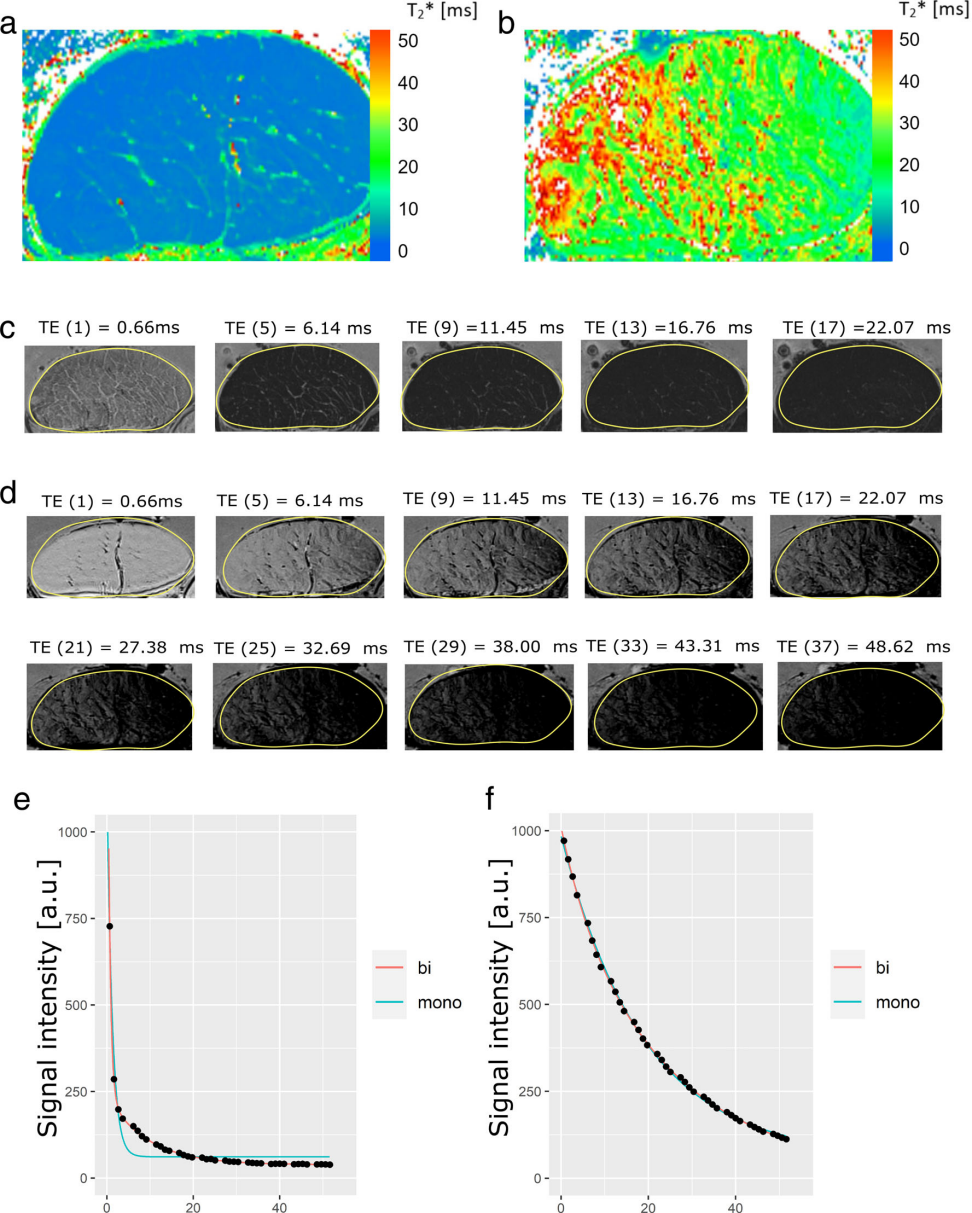
Representative mono‐exponentially calculated T_2_* maps of AT1 measured at a fiber‐to‐field angle of 0° (a) and at 55° (b). T_2_*w images at different echo times at 0° (c) and at 55° (d) are shown. At 0° with an echo time of 22.07 msec no signal is provided from the fascicle tissue but only from nonfascicle parts. In contrast, at 55° the fascicle parts provide signal even at the highest echo time. Both mono‐ and bi‐exponential fitting were performed at 0° and 55°. Representative fits are shown in (e) and (f), respectively. For the other Achilles tendons (AT2, AT3, AT4) measured at the magic angle, the bi‐exponential model was the weaker model compared to the mono‐exponential model.

In this context, it is useful to consider AT1. Figure 2e shows the T_2_* decay and the respective mono and bi‐exponential fitting of AT1 at 0° and 55°. The slice‐wise analysis showed a clear bi‐exponential decay for 0° according to *F*‐test and AIC_C_. Furthermore, for this tendon, the bi‐exponential model was found to be the preferred model for the magic angle according to AIC_C_ and F‐test. While visually it could be seen that the two models fit the data at 55° almost congruently, the bi‐exponential model was the preferred one in this example. For example, the *P* values of the *F*‐test at 55° (*P* < 0.001) for 29 of 30 slices and at 0° for all slices (*P* < 0.001) clearly showed the preference of the bi‐exponential model for AT1.

Since the bi‐exponential fit either did not converge for angles close to the magic angle or was considered the weaker model in many cases, the table fields for which no result could be provided were left blank in Tables 1, 2, 3 accordingly.

**FIGURE 3:**
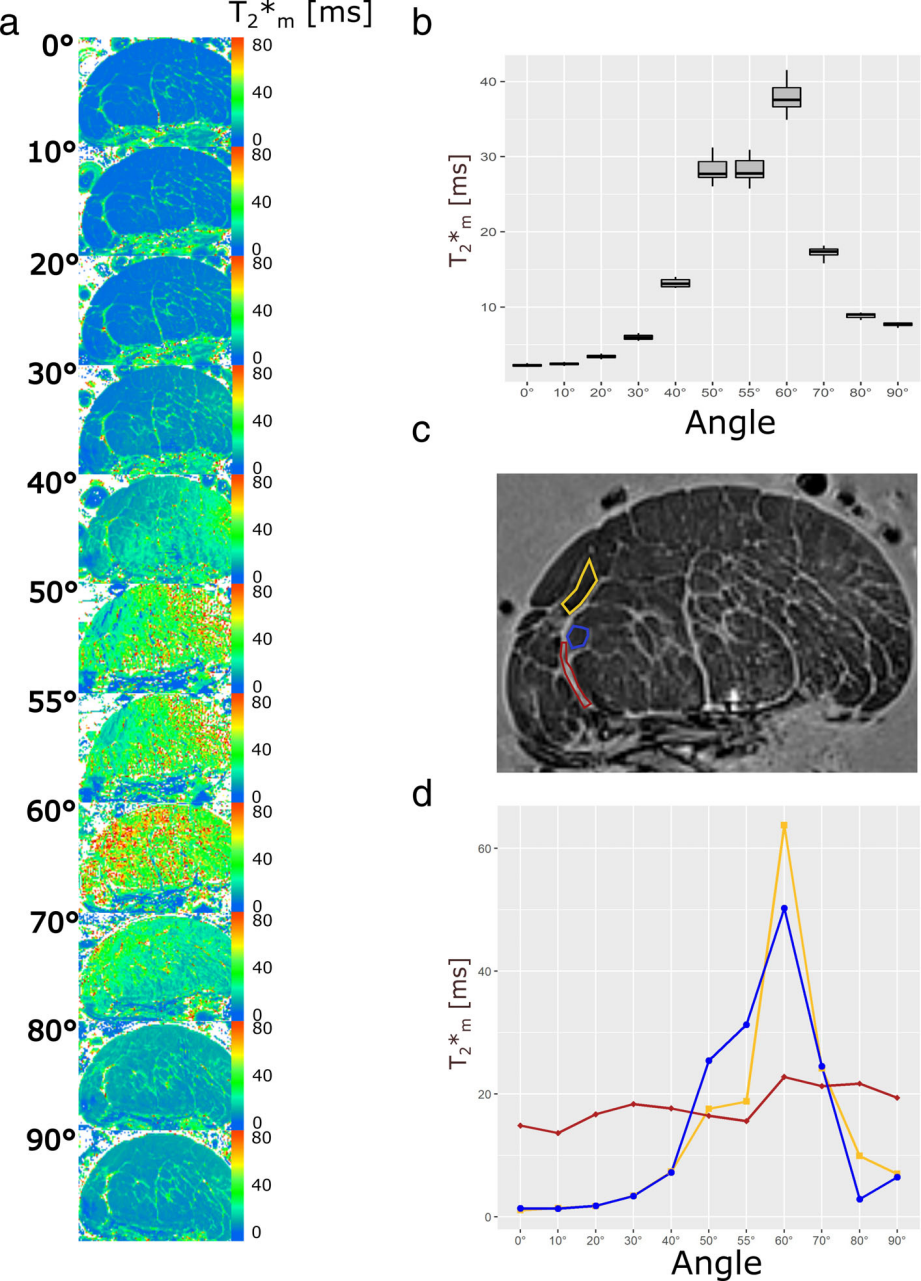
Representative T_2_* maps of the AT4 measured at 11 fiber‐to‐field angles (a). The mono‐exponential T_2_* values increase from 0° toward 55° as is shown in the images and in the boxplot (b), where a slice‐wise analysis of the T_2_* values of 30 slices was performed. Figure 3c shows the position of the selected ROIs for compartment‐specific T_2_* analysis in a T_2_*w image (of the same representative slice as the T_2_* maps). The blue and yellow ROI are set to regions of the polygon‐shaped fascicles, while the red ROI is from nonfascicle tissue. The T_2_* values of the fascicle tissue feature a strong angle dependence. The T_2_* values almost increase 60‐fold for the yellow ROI. Moreover, the T_2_* values of the fascicle tissue reach and even exceed the T_2_* values of the non‐fascicle tissue at angles close to the magic angle (d).

**FIGURE 4:**
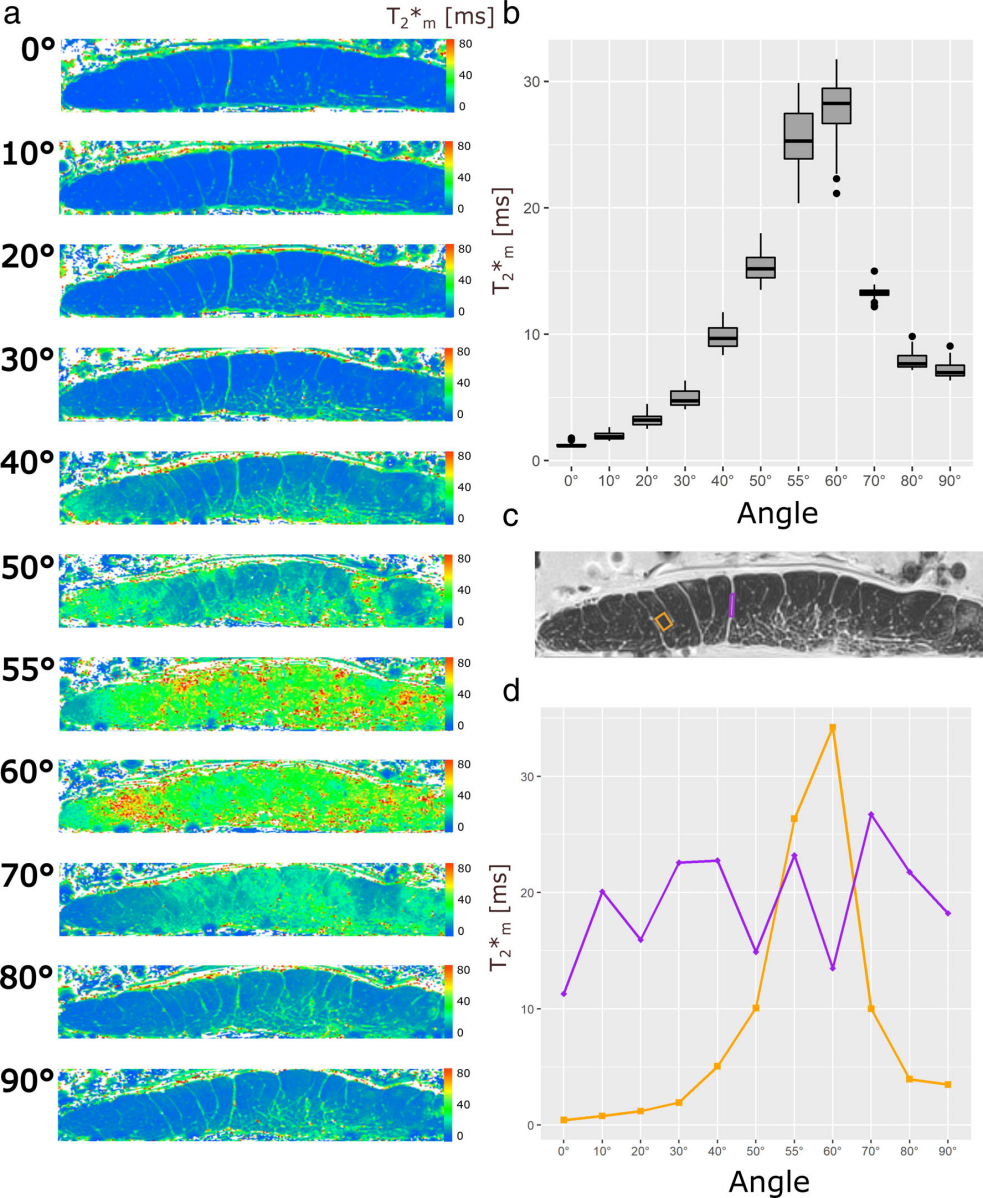
Representative T_2_* maps of the PT4 measured at 11 fiber‐to‐field angles (a). Similar to what is shown in Figure 3 with the AT4, the T_2_* values of the patellar tendon change with angle and reach their maximum T_2_* values at the magic angle of 55° (b). Figure 4c shows the position of the selected ROIs for sub‐tissue‐specific T_2_* analysis in a T_2_*w image (TE = 6.14 msec, fiber‐to‐field angle = 20°). The orange ROI is placed in the fascicle tissue, whereas the purple ROI is positioned in the nonfascicle tissue. The T_2_* values of the orange fascicle ROI increase almost 80‐fold from 0° toward the magic angle, and the T_2_* values of the fascicle ROI also exceed the T_2_* values of the nonfascicle tissue at the magic angle (d).

T_2_* maps of AT4 and PT4 at 11 angles to the static magnetic field are presented in Figs. 3a and 4a, respectively. The boxplots of the slice‐wise calculated T_2_* values are presented in Figs. 3b and 4b. The whole slice zoomed to the area of the tendon covered about 10,000 voxels in each case. Tables 3, 4 and Fig. 3a,b present the results from the same Achilles and patellar tendon measured at 11 angles. The results show the alterations of the T_2_* values with fiber‐to‐field angle with a minimum at 0° (T_2_*_m_ = 2.26 msec for AT4 and T_2_*_m_ = 1.25 msec for PT4 at 0°) and a maximum at angles close to the magic angle (T_2_*_m_ = 37.93 msec of AT4 and T_2_*_m_ = 27.74 msec of PT4 at 60°). The T_2_* values then decrease again to some extent up to 90° (T_2_*_m_ = 7.71 msec of AT4 and T_2_*_m_ = 7.17 msec of PT4 at 90°).

For AT4, we found that T_2_*_m_ values change on average by a factor of 16.78 when comparing angles reflecting minimum and maximum dipolar interactions (Table 3 and Fig. 3a). While the T_2_* decay is clearly bi‐exponential at angles around 0° according to *F*‐test and AIC_C_, this aspect gradually decreases at angles toward 55°. At angles of 50–70°, no physically meaningful bi‐exponential fit could be performed (as described in the Methods and Materials section). At 80° and 90°, the T_2_* decay is not unambiguously mono or bi‐exponential. The T_2_* decay of some slices is preferably mono‐exponential and for some slices it is preferably bi‐exponential according to AIC_C_ and *F*‐test. Accordingly, the results of the bi‐exponential analysis are given in parentheses because they do not reflect the results of all 30 slices.

For the PT4, we found that T_2_*_m_ values increased by more a factor of 22.19 on average, when comparing the data of 0° with that of 60° (Table 4 and Fig. 4a,b). Similar to the AT4, the results show a clear bi‐exponential decay at angles close to 0° as well as most other angles (Table 4).

For fascicle ROIs, changing the angle from 0° to 55° increased the T_2_*_m_‐values by up to a factor of 80 from T_2_*_m_ (0°) = 0.43 msec to T_2_*_m_ (55°) = 34.21 msec (Figs. 3c,d and 4c,d). The T_2_*_m_ of the nonfascicle regions ranged approximately between 15 msec and 25 msec at all angles. At 60°, for instance, the T_2_*_m_ values of the fascicle ROIs were T_2_*_m_ = 63.79 msec (AT4, yellow ROI), T_2_*_m_ = 50.28 msec (AT4, blue ROI) and T_2_*_m_ = 34.21 msec (PT4, orange ROI). The T_2_* decay of the fascicle ROIs showed a clear preference for a mono‐exponential decay according to AIC_C_ and *F*‐test in all angles.

## Discussion

In our study, we demonstrated that quantitative T_2_* mapping with microscopic resolution provides more detailed insight into the relationship between T_2_* decay (mono‐ vs. bi‐exponential model), T_2_* anisotropy and tendon heterogeneity. Our results indicate the impact of the substructure of the tendon as a possible source of misinterpretation concerning T_2_* mapping of tendons and its potential use to detect tendon degenerations. A difference of only 10° fiber‐to‐field angle can already change T_2_* values by about 100% (see, e.g., the increase in T_2_* values from 30° to 40°).

Juras et al reported T_2_*_m_ values of T_2_*_m_ = 3.35 msec in healthy volunteers, whereas they were T_2_*_m_ = 6.56 msec in patients with degenerated Achilles tendons.^1^ As the results of our study show, a change in fiber‐to‐field angle of only 10° may already have a similar effect on the T_2_*_m_ values as the difference caused by degeneration. This could complicate the future use of T_2_* mapping to detect degenerative changes in tendon tissue. Many tendons or their fibers are measured at an angle with respect to the magnetic field that can be considered relatively constant. Such a case would be, for example, an Achilles tendon in an ankle coil, where the sole of the foot is usually positioned perpendicular to the magnetic field, which then gives the long axis of the Achilles tendon a fiber‐to‐field angle of approximately 0°. This also explains, for instance, why the Achilles tendon usually provides no signal in morphological MRI and is characterized by extremely short T_2_* values in quantitative MRI.^1^

In contrast, other tendons, such as the supraspinatus tendon are not easily measured at the same angle. The particular fiber‐to‐field angle depends on both the anatomy (i.e. the course of the tendon in the human body) and the positioning of the patient in the respective coil. In any case, it is impossible to always position tendons to the same fiber‐to‐field angle and therefore a possible misinterpretation of the T_2_* values, for example, in terms of a false positive detection of degeneration due to a large fiber‐to‐field angle, is difficult to avoid. However, in the same institution, with the same scanner and coil, the same fiber‐to‐field angle allows defining tendon degeneration in different patients with the same setup. In multicenter studies, if the tendons being compared do not have the same fiber‐to‐field angle with high accuracy, the results cannot be compared with each other since T_2_* anisotropy clearly appears to be a dominant influencing factor in this context.

While the Achilles tendon and the patellar tendon show strong structural similarities,^18^, ^31^ the T_2_* characteristics seem to indicate some differences. In our experiments, we used Achilles tendon samples from the middle section of the Achilles tendons. In these sections, the fibers run mostly parallel to the long axis of the tendon. In contrast, the fibers of the middle section of the patellar tendon are more fan shaped.^31^ This may explain why the T_2_*_m_ values of the patellar tendon at 55° do not reach as high T_2_*_m_ values as those of the Achilles tendons. When a significant portion of the fibers deviate slightly from the direction of the tendon's long axis (to which the experiments are adjusted), there is inevitably some attenuation of the anisotropy effect.

The results of our study also show that the regional differences in the T_2_* values are due to the heterogeneous structure of the tissue. These regional differences have a dominant influence on the resulting T_2_* fiber‐to‐field angle dependence and on deciding which decay model, that is, mono‐ or bi‐exponential decay model, is the preferred one for particular angles. In this context, we would like to point out that a tendon is not a homogeneous entity, even though it may appear so even in high‐resolution MRI studies.^32^ Instead, it consists—with respect to overall volume—of a dominant fascicle part and a nondominant nonfascicle part (including endotenon and paratenon), which are mainly composed of water and proteoglycans.^18^ These noncollagenous components have previously not been taken into consideration in T_2_*‐mapping studies mainly because the spatial resolution of the respective studies was either insufficient or nonexistent (nonlocalized MR measurements) in order to visualize them. However, their possible influence on multicomponent T_2_* decay has been suggested previously.^8^ Indeed, we found that in fascicle regions, the T_2_* anisotropy was extremely pronounced, while in the nonfascicle regions the T_2_* values seem to be hardly affected by the angle, which was to be expected since the water molecules in such sub‐tissues are presumably significantly less restricted. For the whole slice, the maximum increase in T_2_* is less than in fascicle regions alone, because in the whole slice both sub‐tissues (fascicle and nonfascicle tissues) are combined.

For the tendons as a whole (this was selected by means of ROIs covering the whole tendon in the axial direction), we found that the tendons at angles 0°, 10°, 20°, and 30° show clear bi‐exponential T_2_* decay, while for angles closer to the magic angle, the mono‐exponential decay model was predominantly found by *F*‐test and AIC_C_ as the preferred model over the bi‐exponential model.

There is a relatively simple explanation for this behavior, as shown by our ROI analysis: In the range from 0° to 30°, the fascicle compartment of the tendon yielded very short to short T_2_* values and was thus easily distinguished from the higher T_2_* values of the surrounding nonfascicle portion. Therefore, the T_2_* decay of the entire tendon, or at voxel sizes commonly used in whole‐body MRI, appeared to be clearly better described by a bi‐exponential model at such angles.

An advanced model should consider that not all fascicles of a tendon run in exactly the same direction, which also means that when we look at the axial cross section of a tendon, the T_2_* values of the fascicle component correspond to a distribution of T_2_* values rather than a uniform value as can be clearly seen in the presented T_2_* maps. In some areas, the T_2_* values may be lower, because the fibers there exhibit a rather low angle with the magnetic field, while in another part they are oriented somewhat closer to 55° and this part may therefore feature longer T_2_* values. When considering the whole tendon again, by changing the angle from 0° toward 55°, one can imagine that with each small change in angle, the short component is brought closer and closer to the long component. Our ROI analysis suggests that the fascicle component, which is predominantly responsible for the short component of T_2_* at 0°, changes dramatically with angle, while the nonfascicle component appears to be rather unaffected by angle changes. When the angle is shifted toward 55°, the short and the long components of T_2_* change as a consequence of these mechanisms. The component ratios flip over at 30°–40°. The longer component becomes more substantial than the short one. With even higher angles towards 55°, the short component approaches the values of the long component until it is no longer distinguishable and as a result, the T_2_* decay appears as mono‐exponential decay.

Reviewing previous theoretical and molecular considerations on the molecular dynamics in tendons explaining the known properties of T_2_ and T_2_* decay in the magnetic field,^2^, ^33^ it is reasonable to assume that these considerations referred purely to the fascicle part of the tendon, where the water molecules are restricted in their rotational and translational mobility. On the contrary, however, we can safely assume that water molecules in nonfascicular tissues such as the endotenon is much more unrestricted in their rotational and translational mobility and dipolar interactions can average out there.

This is also reflected in our results. The T_2_* decay of nonfascicle regions appears to be hardly angle dependent, while the fascicles themselves show a change by a factor up to 80 when measured between 0° and 55°. Minor angular T_2_* changes between 0° and 55° that we observed in the small ROI analyses of the nonfascicle regions were probably due to partial volume effects (voxels containing partially fascicle tissue), after all sub‐tissue such as the endotenon is very thin. However, further measurements at even higher in‐plane resolution and thinner slices are needed in this context.

In the Introduction, it was mentioned that the collagen fibers of the tendon are oriented approximately along the longitudinal axis of the tendon. However, this is only an approximation. In the Achilles tendon, for example, the collagen bundles run from the calcaneus toward the gastrocnemius and soleus muscles in a twisted manner.^34^ Thus, it is not surprising that in our experiment, in which we measured an AT at 11 angles, the fiber bundles on one side of the T_2_* map of the tendon cross section seem to reach the actual magic angle best at 55° (resp. reach the highest T_2_* values there), while the other side of the T_2_* map of the tendon cross section seems to be further away from it, but it reaches the highest T_2_* values at 60°, that is, the fiber bundles on this side of the tendon cross section were then obviously closer to the actual magic angle.

Similarly, the Achilles tendon (AT1) does not appear to be perfectly oriented at a 55° angle. While on the left side of the tendon, the fiber bundles might have been reached the magic angle, the right side appears to be rather in the angle of approximately 30°, which also explains its shorter T_2_* values and faster signal decay there. In addition, this explains why this tendon features a preferential bi‐exponential decay at this angle. However, the comparison of mono‐ and bi‐exponential fitting shows that the two fitting methods can hardly be distinguished here. Which also raises the question as to when a decay should still be considered bi‐exponential.

However, for AT2, AT3, and AT4 either a physically meaningful bi‐exponential fit could not be performed, or the bi‐exponential model proved inferior to the mono‐exponential model according to AIC_C_ and *F*‐test.

At the magic angle, the contrast between fascicle bundles and endotenon is not well visible in microscopic T_2_* maps. This can be explained, as already mentioned earlier, by the fact that the T_2_* values of the fascicle portion in the magic angle region reach extremely high T_2_* values closer to or even exceeding the T_2_* values of the endotenon, which again explains why the T_2_* decay of the tendons in a slice‐by‐slice analysis is preferably mono‐exponential at angles close to the magic angle. This feature has also been found in previous studies on other collagen tissue.^35^ The reasons for this characteristic have only been speculated upon,^35^, ^36^ but a conclusive explanation has not yet been presented.

A multiexponential character with up to around four well‐defined components of the transverse relaxation decay was found in nonlocalized NMR spectroscopy.^8^, ^13^

The assignment of the short and long T_2_ and T_2_* components to bound and free water pools has been repeatedly used in the literature,^1^, ^7^ representing a simplification with regard to the complex substructure of the tendon. As an example, the water surrounding the collagen fibers is not bound, but it is restricted in its translational and rotational mobility.^2^, ^4^ The water in the nonfascicular part of the tendon, on the other hand, is likely to be considered relatively free in its mobility. As such, this assignment is partially consistent with our results. At least for a fiber–field angle of 0°, it seems to hold that the restricted mobility of water protons along the collagen fibers in the fascicles leads to the short component of T_2_*, whereas free water pools in the region outside the fascicles cause the long component of T_2_*. It is noteworthy, however, that this statement is no longer true for angles above approximately 40°, as the fascicle region obtains the same of even higher T_2_* values than the nonfascicle regions.

The argument could be made that the use of a vTE sequence compared with a radial UTE sequence could leave a very short T_2_* compartment undetected, for example, originating from protons of the collagen itself. However, at least in a previous study by Ma et al^37^ using a radial ultrashort TE sequence, it was shown that protons of the native collagen could not be directly visualized even with TEs down to 8 μsec.

The results in our study do not contradict previous results such as those of Juras et al or Liu et al,^1^, ^7^ which showed that certain voxels of a tendon or the whole tendon can be considered bi‐exponential especially at angles close to 0°, but rather provide additional insight on the origin of this observed bi‐exponential decay. Our present study demonstrates that voxels combining both types of tissue compartments (fascicle and nonfascicle components) could explain the bi‐exponential T_2_* decay found in these earlier studies.

There are also other attempts to explain the source of the respective T_2_* components. It is known from theory that residual coupling alone may cause bi‐exponential decay, as shown in the work of Kruk et al.^38^ In addition, large‐scale collagen crimping domains at different angles with respect to B_0_ have been suggested as a possible source of multicomponent T_2_* decay.^5^ Our results suggest that heterogeneity of the tissue may play an important role, but it is very likely that a combination of different mechanisms is ultimately involved in tendon bi‐component T_2_* characteristics and anisotropy properties, and more research is needed to reach a clear conclusion about the origin of bi‐exponential T_2_* decay.

### Limitations

In this study, only a small sample size was used, which may lead to some bias. However, these samples were measured with extremely high accuracy and long measurement time and the results were unambiguous and statistically significant.

The echo time range can be seen as another limitation. At a fiber‐to‐field angle of 0°, the short T_2_* component may have been sampled with only the first three or four echo times, which could have led to minimal uncertainty in the calculation of the short component.

### Conclusion

Our study demonstrated that microscopic T_2_* mapping of tendons provides a deeper understanding of the relationship between T_2_* decay characteristics (mono‐ vs. bi‐exponential decay), T_2_* anisotropy and the heterogeneous structure of tendons.

## Supporting information


**Appendix S1**: Supporting Information
