# Supplementary material for: Transverse Relaxation Anisotropy of the Achilles and Patellar Tendon Studied by MR Microscopy
Source: J Magn Reson Imaging. 2022 Feb 5;56(4):1091–103. doi: 10.1002/jmri.28095 (PMC9545006; doi:10.1002/jmri.28095)
Supplement: Supplementary file 1 — Appendix S1: Supporting Information [file JMRI-56-1091-s001.docx]

# **Supplementary section**

# **Dipolar intramolecular interaction of protons in collagen-rich tissues**

The dipolar interaction between two spins *j* and *k*, can be either intranuclear (i.e., considering the protons of water, the interaction of a proton of one water molecule with the other proton of the same water molecule) - or internuclear (i.e., the interaction of protons of different water molecules). The magnitude of the respective interaction through space can be given by the dipole-dipole coupling constant as follows ([1](#_ENREF_1)):

|  | $g_{jk}=-\frac{\mu_{0}}{4\pi} \frac{\gamma_{j}\gamma_{k}\hbar}{r_{jk}^{3}}$ | (1) |
| --- | --- | --- |

where r*_jk_* refers to the distance between the two spins, $\gamma_{j}$ and $\gamma_{k}$ to their gyromagnetic ratios (42 MHz T^-1^ for each proton), $\mu_{0}$ to the magnetic constant (*µ*_0_ = 4π x 10^-7^ H m^-1^) and *ħ* to the Planck constant (*ħ* = 1.054571817 x 10^-34^ J s).

The truncated secular (i.e. the time-independent) Hamiltonian can be given as follows ([1](#_ENREF_1)):

|  | ${\hat{\mathbf{H}}}_{jk}^{\mathrm{DD}}(\theta_{jk})= d_{jk}(3 \hat{I}_{jz}\hat{I}_{kz} {- \hat{\mathbf{I}}}_{j}\cdot{\hat{\mathbf{I}}}_{k})$ | (2) |
| --- | --- | --- |

where ${\hat{\mathbf{H}}}_{jk}^{\mathrm{DD}}$ is the Hamiltonian operator of the dipolar interaction. The angle $\theta_{jk}$ refers to the angle between the vector connecting the interacting spins and the vector of the magnetic field and $d_{jk}$ refers to the secular dipole-dipole coupling, which can be given as follows ([1](#_ENREF_1)):

|  | $d_{jk}= \frac{1}{2} g_{jk}\sqrt{3 \cos^{2} \theta_{jk}-1}$ | (3) |
| --- | --- | --- |

The expression $3\cos^{2} \theta_{jk}-1$ is the second order Legendre polynomial. The secular dipole-dipole coupling $d_{jk}$ becomes zero if $3\cos^{2} \theta_{jk}-1$becomes zero ([1](#_ENREF_1)):

|  | $3\cos^{2} \theta_{jk}-1=0$ | (4) |
| --- | --- | --- |

The angle $\theta_{jk}$ that satisfies this equation is the so-called magic angle $\theta_{m}$ ([1](#_ENREF_1)):

|  | $\theta_{m}=arctan \sqrt{2} \cong54.74^{\circ}$ | (5) |
| --- | --- | --- |

It is well known, that the collagen fibers in collagenous tissue are surrounded by water molecules, and it is predominantly from the protons of these water molecules that a signal can be obtained in MRI of collagen tissue.

In general, it was found that the distance between two protons in a water molecule is approximately 1.5-1.6 Å (10^-10^ m), as shown by ^17^O NMR spectroscopy and neutron diffraction ([2](#_ENREF_2),[3](#_ENREF_3)), while the distance of a proton from one water molecule to the next (intermolecular ^1^H-^1^H distance) is considerably larger, with at least 2.2 Å, owing to Van der Waals contact forces ([4](#_ENREF_4)).

The dipolar interaction decreases with the inverse third power of the inter-/intraproton distance r_jk_ for the case where the protons involved are restricted in their rotation, as is also the case for water molecules surrounding collagen fibers. Apart from this, in the absence of rotational constraint as in a bulk solution, the dipolar interaction is assumed to decrease with the inverse sixth power of the distance ([5](#_ENREF_5)).

Considering the intramolecular and intermolecular ^1^H - ^1^H distances mentioned above, and taking into account that dipolar coupling depends inversely on proton distance, the dipole-dipole interaction that we experience in MRI of collagen-rich tissue is thought to occur almost exclusively through intramolecular interactions rather than intermolecular interactions ([6](#_ENREF_6),[7](#_ENREF_7)).

Coming back to the collagen fibers in the collagen tissue which are surrounded by water molecules; molecular dynamic simulations have shown that the water molecule director(s) (i.e., the direction of the symmetry axis of the water molecule) are each perpendicular to the fiber(s) ([6](#_ENREF_6),[7](#_ENREF_7)). Thus, an ensemble-averaged intramolecular vector is oriented in the same direction as the fiber. Therefore, in a tissue such as a tendon, where the fibers are oriented approximately parallel to the longitudinal axis of the tendon, the ensemble-averaged intramolecular $\left\langle{}^{1}H-\left. {}^{1}H \right\rangle\right.$vector is therefore also approximately oriented along the longitudinal axis of the tendon ([7](#_ENREF_7)) (Supplementary figure. 1), which is ultimately responsible for the orientation-dependent T_2_* properties of a tendon.

For example, according to equation 3, if the ensemble-averaged intraproton vector is aligned with the direction of the main magnetic field ($\theta=0$), then the dipolar interaction is at a maximum, leading to significant frequency and phase shifts and hence very short transverse relaxation times (T_2_ and T_2_*). In contrast, for the magic angle, the transverse relaxation times are the longest because the dipolar interaction is lowest there (strictly speaking zero at the magic angle). Therefore, depending on the angle *θ*, there is a variability of the intramolecular dipolar interaction of the protons in the collagen tissue which is reflected in corresponding changes of transverse relaxation times.

**References:**

1. Levitt MH. Spin dynamics: basics of nuclear magnetic resonance. Chichester, UK: John Wiley & Sons: 2001.

2. Keeler EG, Michaelis VK, Griffin RG. 17O NMR investigation of water structure and dynamics. The Journal of Physical Chemistry B 2016;120(32):7851-7858.

3. Sikka S, Momin S, Rajagopal H, Chidambaram R. Neutron‐Diffraction Refinement of the Crystal Structure of Barium Chlorate Monohydrate Ba (ClO3) 2· H2O. The Journal of Chemical Physics 1968;48(5):1883-1889.

4. Van-Quynh A, Willson S, Bryant RG. Protein reorientation and bound water molecules measured by 1H magnetic spin-lattice relaxation. Biophysical journal 2003;84(1):558-563.

5. Solomon I. Relaxation processes in a system of two spins. Physical Review 1955;99(2):559.

6. Tourell MC, Momot KI. Molecular dynamics of a hydrated collagen peptide: insights into rotational motion and residence times of single-water bridges in collagen. The Journal of Physical Chemistry B 2016;120(49):12432-12443.

7. Momot KI, Pope JM, Wellard RM. Anisotropy of spin relaxation of water protons in cartilage and tendon. NMR in Biomedicine 2010;23(3):313-324.

**Supplementary figure 1**: Schematic representation of the water molecules surrounding the collagen fibers and the intramolecular dipolar interaction of protons. The individual intramolecular vectors are depicted. Furthermore, it is shown that the ensemble averaged intramolecular vector is oriented in the same direction as the fiber. Reproduced with permission from Tourell et al ([6](#_ENREF_6)).
